# Clinical Remarks upon Surgical Cases in the Buffalo General Hospital—Gun-Shot Wound, Amputation at the Shoulder-Joint—Exsection of the Radius—Necrosis after Amputation, Remarkable Reproduction of Bone

**Published:** 1865-02

**Authors:** J. F. Miner


ART. III.—*Clinical Remarks upon Surgical Cases in the Buffalo General Hospital—Gun-shot wound, amputation at the shoulder-joint—Exsection of the Radius—Necrosis after Amputation, remarkable reproduction of bone.* By J. F. Miner, M. D.

November, 15, 1864.

Gentlemen:

The young lad I present before you this morning, has been so unfortunate as to have discharged the contents of a musket, loaded with pistol balls, buck-shot, etc., through the upper portion of his right arm. It has broken in fragments the upper part of the humerus, and has lacerated the muscles of the arm very extensively. The brachial artery is as yet unbroken, while everything near it on all sides is destroyed, the charge having passed from directly opposite the principal vessels through the arm, by some wonderful guidance not cutting this artery. Hemorrhage has been considerable from the extensive laceration of small vessels, and the arm as you observe appears already about half amputated by the accident.

With the advice of my colleagues and their assistance, I propose to amputate at the shoulder-joint, not however, without having considered the propriety of making an exsection and removal of the fragments of bone, or of removing the loose bones, dressing the arm as in fracture, and awaiting the issue. It is proper that I tell you the objections to these apparently more conservative operations, and let you know what has influenced our course of action. If this arm should be dressed as a fracture simply, first removing the loose fragments of detached bone, what in all human probability would be the result? To answer this question briefly, I should say 1st—Continued exhausting hemorrhage from the immense surface of laceration. 2d—Rupture of the brachial artery, the injured and unsupported walls of the vessel giving way before the progress of the ulceration which is likely to follow. 3d—An arm with ununited fracture, with nerves destroyed and thus doubly paralyzed, could hardly compensate for the great risk which such a course would involve—risk of hemorrhage, suppuration, absorption of pus, and all the other dangers of such severe injuries.

Exsection is obnoxious to the same objections; it has too little to offer and too much to risk. The bone is fractured too high up—the large vessels are too much endangered, the process of repair is too slow and uncertain, and if at last all the dangers are safely passed, there is at best but little gained; an arm without a bone, without sensation, and without useful motion, is of no value for such a boy—is not worth risking a single chance of his life, to preserve.

When we come to consider the arguments in favor of the operation I propose to make, there are still dangers, uncertainties and risks. Amputation at the shoulder-joint after injury, is a very dangerous operation to have made; it is easy enough to make, but not highly successful in its results; it can never be made without greatly endangering the life of the patient; with these sources of danger you are all sufficiently familiar. It appears to us as the only way to save this boy's life, and yet we are fully conscious of its uncertainties and of the fearful risks to which he is exposed. The operation itself I have no occasion to describe; it is varied from the usual plan only in making my first flap more posteriorly; this I am obliged to do on account of the extensive laceration which prevents the use for this purpose of the deltoid muscle and integuments covering that region. This however is no inconvenience, and you now observe that the arm is removed with great ease at the shoulder-joint, and that we have perfect adaptation of the parts, though the flaps are made posteriorly and anteriorly. This plan of operation is practiced by some surgeons from choice; in this instance we have adopted it from necessity; perhaps it is as well as any.

Our 2d operation is for removal of the necrosed portion of the radius. Our patient received gun-shot wound about eight months since, the radius being fractured and comminuted extensively. We now detect necrosed bone, and there is no prospect of union or recovery, at least for the present, without its removal. We therefore exsect the dead portion entire, leaving a space of about four inches to be filled by whatever material nature may supply. It is quite remarkable what wonderful provisions nature sometimes makes for the repair of similar injuries. She cannot be relied upon to certainly provide a bony union or connection, but when the periostium is left entire nature sometimes restores similar losses by bone; we have reproduction of bony tissue under favorable circumstances, but it is rare, and not to be expected. A cartilaginous or fibro-cartilaginous connection is however to be depended upon. A favorable result then in this case will be recovery without bony union. Our patient will be entitled to his discharge from military duty after the most favorable result.

We have one other operation for removal of dead bone. This young soldier had his arm amputated about one year since; there is dead bone now protruding, not covered by integument. This discharge of pus is very great; the system depressed by this discharge. He is also suffering from what is commonly called "chronic diarrhœa." I advise the removal of this dead bone, as the only way of arresting the diarrhœa; other physicians have not looked upon his case in that light, and have tried to stop the discharge from the bowels first, deferring the removal of dead bone until after recovery. I think his diarrhœa will abate when the system has rallied, as it will upon the cessation of this immense purulent secretion, and as a remedy for all his troubles I propose to remove this dead bone.

Upon cutting down upon this, we find that a new bone has grown around the dead one, and that the whole of the upper third of the humerus has become necrosed after amputation, and I have extracted it almost entire from the bony case which has formed around it. The exact condition had not been mistrusted until this operation has shown us the condition of things. It is much of the nature of necrosis without amputation; new bone has grown around the old and dead one, the cloca leading to it is at the end, and in this case is sufficiently large to allow extraction of the dead portion. It is a very remarkable case of reproduction of bone; so rare that you will observe it probably but once, in your lives.

Necrosis of the stump after amputation is common, but death of the whole bone and reproduction after the manner you here observe is a termination so rare and remarkable as to be worthy of rememberance.*

* The cases above described have terminated very favorably; the amputation at shoulder joint recovered without accident or delay. Hospital gangrene attacked the arm of the second, but after ten or twelve days healthy granulations commenced and recovery is very sasfactory. Recovery of the last has been astonishingly rapid. Healthy granulations have filled the cavity left by extraction of bone. Diarrhœa ceased, and flesh and strength have been fully gained.

